# Assessment of Vertical/Horizontal Ridge Augmentation in Atrophic Alveolar Ridge Using Autogenous Onlay Versus Inlay Grafting Techniques: A Systematic Review

**DOI:** 10.7759/cureus.100809

**Published:** 2026-01-05

**Authors:** Harshda S Mahajan, Rajesh Gaikwad, Akshaya Banodkar, Shubham B Tale, Madhumitha Chidambaram, Monisha Iyer, Sanpreet S Sachdev

**Affiliations:** 1 Department of Periodontology, Government Dental College and Hospital, Mumbai, Mumbai, IND; 2 Oral Pathology and Microbiology, Bharati Vidyapeeth (Deemed to be University) Dental College and Hospital, Navi Mumbai, IND

**Keywords:** atrophic ridges, inlay grafting technique, jawbones, onlay grafting technique, ridge augmentation procedures

## Abstract

Alveolar ridge atrophy presents a major challenge in implant dentistry, often necessitating surgical augmentation to restore adequate bone volume for predictable implant placement. Among the various autogenous grafting approaches, the inlay and onlay techniques are widely utilized, yet their comparative clinical efficacy remains unclear. This systematic review aimed to evaluate the outcomes of vertical and horizontal ridge augmentation using inlay versus onlay grafting techniques in atrophic alveolar ridges. An extensive electronic and manual search of major databases was conducted in accordance with PRISMA guidelines, and eligible randomized controlled trials and comparative clinical studies assessing autogenous grafts were included. Six studies met the inclusion criteria. Both techniques produced appreciable gains in bone height and width; however, the inlay technique demonstrated more favorable results in terms of graft stability, reduced volumetric resorption, and improved soft tissue healing. Inlay grafts benefited from superior vascularization and mechanical protection due to their placement within the osteotomized host bone, resulting in fewer complications and better preservation of augmented volume. By contrast, onlay grafts showed higher rates of resorption, flap dehiscence, and graft exposure, particularly in vertical augmentations where soft tissue tension and limited vascular supply are significant challenges. Despite promising findings, heterogeneity among studies and limited long-term data restrict definitive conclusions. Further well-designed randomized controlled trials with standardized protocols and extended follow-up are needed to strengthen the evidence base. Overall, the inlay technique appears to offer predictable and stable outcomes, particularly in complex augmentation scenarios.

## Introduction and background

Alveolar ridge atrophy remains a significant clinical challenge in dentistry and maxillofacial surgery, particularly when planning implant-supported or prosthodontic rehabilitation in edentulous regions. Following tooth loss, the alveolar bone undergoes a continuous and progressive resorptive process, resulting in a substantial reduction in vertical height and horizontal width of the ridge [1,2]. This resorption is most rapid during the first year after extraction but continues, albeit at a slower pace, throughout life [3]. Multiple etiologic factors contribute to ridge atrophy, including trauma, infection, periodontal disease, congenital anomalies, and extended periods of edentulism [4,5]. In addition to local and pathological factors, underlying skeletal discrepancies also play a crucial role in determining alveolar bone volume and ridge morphology. Variations in sagittal jaw relationships, such as those observed between skeletal Class I and Class III patterns, have been shown to influence baseline jawbone dimensions and the severity of alveolar deficiencies. Patients with skeletal Class III patterns may present with altered mandibular and maxillary bone volumes, potentially necessitating more complex augmentation strategies to achieve prosthetically driven implant placement [6]. Recognizing these anatomical variations is essential for individualized treatment planning and for selecting appropriate ridge augmentation techniques. 

The resulting deficiency compromises prosthetic stability, esthetics, and functional outcomes, often necessitating surgical augmentation procedures before implant placement [7]. Thus, re-establishing adequate alveolar bone volume becomes a prerequisite for achieving predictable osseointegration and long-term prosthetic success [8,9]. Autogenous bone grafting is widely regarded as the gold standard for ridge augmentation due to its inherent osteogenic, osteoconductive, and osteoinductive properties [10]. Among the various surgical strategies, onlay and inlay grafting techniques are two established autogenous approaches used to restore deficient alveolar ridges [11]. These methods differ in biological behavior, mechanical stability, surgical indications, and long-term performance [11,12]. Onlay grafting involves placement of a bone block or particulate graft on the external surface of the alveolar ridge to increase height or width [13]. It is often used in cases with generalized vertical or horizontal bone deficiency and typically requires rigid fixation to ensure stability and intimate graft-host contact [12,13]. Successful onlay augmentation depends heavily on adequate revascularization through the host bed and periosteum, rendering the technique sensitive to surgical handling and flap management [14].

By contrast, the inlay grafting technique, although less commonly employed, offers unique biomechanical and biological advantages in selected clinical scenarios [15]. This method involves preparation of a recipient site within the existing alveolar ridge, such as a slot or tunnel, into which the graft is inserted [16]. By positioning the graft within a three-dimensional host bone enclosure, the technique promotes superior vascularization, mechanical stability, and potentially reduced graft resorption [17]. Inlay grafting is particularly indicated for localized or saddle-type defects where the anatomy supports containment of the graft [15-17].

Both techniques have demonstrated clinical success in restoring deficient ridges; however, each is associated with notable limitations. Onlay grafts may undergo significant volumetric resorption, require longer healing, and present higher risks of dehiscence or exposure, particularly in vertically augmented sites [18,19]. Inlay grafts offer better mechanical protection and vascular supply but are more technique-sensitive and limited by anatomical constraints [20]. Owing to these variations, the choice between onlay and inlay grafting depends on defect morphology, surgeon expertise, functional demands, and long-term prosthetic objectives [21,22].

Despite widespread use, a definitive consensus regarding the comparative clinical efficacy of these techniques is lacking, with available studies differing in design, graft type, measurement methods, and follow-up duration. Therefore, a systematic evaluation of current evidence is essential to guide clinical decision-making and improve outcomes in the reconstruction of atrophic alveolar ridges. The present systematic review aims to comprehensively compare the clinical outcomes of Ridge augmentation using the inlay versus the onlay grafting technique.

## Review

Methodology

Study Design

The present systematic review was conducted in accordance with the Preferred Reporting Items for Systematic Reviews and Meta-Analyses (PRISMA) guidelines [23], and the protocol was prospectively registered in the PROSPERO database (Registration ID: CRD42024626052).

Review Question

The primary review question guiding this systematic evaluation was: “Is the inlay grafting technique more effective than the onlay grafting technique in augmenting vertical or horizontal deficiencies of the atrophic alveolar ridge?” This question was developed to compare the clinical performance of both techniques in terms of hard-tissue regeneration and postoperative outcomes and to determine whether one method demonstrated superior predictability in restoring deficient alveolar ridges. The question was framed using the PICOS format: Population (adults ≥18 years with atrophic alveolar ridges requiring augmentation), Intervention (inlay/interpositional grafting), Comparator (onlay grafting), Outcomes (radiographic bone height/width gain, graft resorption/volume maintenance, soft-tissue healing and complications, and implant-related outcomes where reported), and Study design (randomized controlled trials (RCTs) and comparative clinical studies, including prospective and retrospective observational studies with a direct inlay-onlay comparison).

Eligibility Criteria

Prior to the literature search, well-defined inclusion and exclusion criteria were established to maintain consistency and ensure relevance. RCTs and comparative clinical studies (prospective cohorts and retrospective comparative studies) involving human participants aged ≥18 years and directly comparing inlay versus onlay ridge augmentation were considered eligible. Studies had to evaluate autogenous bone grafts placed using either the inlay or onlay technique and provide measurable outcomes, such as bone height or width gain, graft resorption, or soft-tissue healing. Full-text publications written in English and presenting original clinical data were included. Studies involving animal models, in vitro experiments, case reports, case series, narrative or systematic reviews, and investigations using synthetic substitutes were excluded. Studies in which patients presented with systemic conditions or medication use known to influence bone metabolism, as well as studies involving previously augmented sites, were also omitted to avoid confounding factors.

Information Sources

A comprehensive and systematic electronic search was conducted across multiple databases to ensure thorough coverage of the available literature. The databases included PubMed, MEDLINE, Embase, EBSCO, the Cochrane Central Register of Controlled Trials (CENTRAL), Web of Science, and Google Scholar. To broaden the search and reduce the possibility of missing relevant studies, the reference lists of all eligible and near-eligible articles were manually screened. No restrictions were applied regarding the year of publication to allow inclusion of all potentially relevant historical and contemporary evidence. Only studies published in English were considered.

Search Strategy

A structured and reproducible search strategy was developed using combinations of controlled vocabulary terms and free-text keywords related to ridge augmentation and grafting techniques. Since all keywords related to the topic that could sufficiently identify all the relevant articles in the search could not be covered using MeSH terms, a combination with free-text keywords was adopted. Commonly used keywords included “inlay grafting technique,” “onlay grafting technique,” “ridge augmentation,” and “atrophic alveolar ridge.” Boolean operators such as AND and OR were used to refine and combine search terms appropriately. The search strategy was applied consistently across all databases to minimize selection bias and ensure comparability. The full electronic search strategy was developed using database-specific controlled vocabulary (e.g., MeSH in PubMed) combined with free-text keywords and synonyms. The complete search strings for each database, including MeSH terms and Boolean operators, are provided in the Appendix.

Study Selection

All retrieved records were screened in two phases. Initially, titles and abstracts were reviewed independently by two investigators to exclude irrelevant and duplicate studies. The screening process was optimized using the Elicit systematic review tool, in which the search results were imported, duplicates were removed, and each item was assigned a screening score based on fulfillment of the abovementioned PICOS criteria. Articles that met the preliminary criteria or lacked sufficient information in the abstract proceeded to full-text screening. In the second phase, full texts were assessed in detail against the predefined eligibility criteria. Any disagreements regarding study eligibility were resolved through mutual discussion. When consensus could not be reached, a third reviewer (RG) adjudicated the final decision.

Data Extraction

Data from the included studies were extracted systematically using a standardized data extraction form. Two reviewers (HM and AB) independently recorded key details such as author names, publication year, country, study design, sample size, demographic characteristics, inclusion and exclusion criteria, type of grafting technique evaluated, duration of follow-up, and clinical outcomes. Extracted outcomes included changes in bone height and width assessed radiographically, graft resorption rates, and qualitative observations of soft-tissue healing. A third reviewer (RG) verified the completeness and accuracy of all extracted data. This tri-level validation minimized errors and ensured that the dataset accurately reflected the original studies.

Outcome Measures

The primary outcomes of interest were the measurable gain in bone height and bone width achieved through the respective grafting procedures, as determined by radiographic imaging modalities such as CBCT. Secondary outcomes included the degree of graft resorption and the quality of soft tissue healing at the grafted site. These parameters were selected for their direct relevance to determining the clinical success and stability of ridge augmentation procedures and their implications for subsequent implant prosthetic rehabilitation.

Risk-of-Bias Assessment

The methodological quality and risk of bias of the included studies were rigorously assessed using established appraisal tools appropriate to each study design. RCTs were evaluated using the Cochrane Risk of Bias 2.0 tool, which assesses domains related to randomization, deviations from intended interventions, missing outcome data, outcome measurement, and selective reporting [24]. Non-randomized clinical studies were appraised using the ROBINS-I tool, which assesses bias arising from confounding, participant selection, intervention classification, deviations from intended interventions, missing data, outcome measurement, and selective reporting [25]. Observational studies, where present, were assessed using the Newcastle-Ottawa Scale [26]. Two reviewers (HM and AB) independently appraised each study, and discrepancies were resolved through discussion or consultation with a third reviewer (RG).

Data Synthesis and Analysis

A qualitative narrative synthesis was planned initially due to anticipated variability in study design, grafting techniques, and outcome reporting. A meta-analysis was planned for studies demonstrating methodological homogeneity and consistently reporting comparable outcomes, such as bone height or bone width gain. Confidence intervals were reported when explicitly provided in the original studies; however, consistent reporting of the confidence intervals was not possible due to heterogeneity in outcome presentation across included trials. Continuous data were intended to be pooled using mean differences with 95% confidence intervals. Heterogeneity across studies was to be assessed using the I² statistic. Statistical analyses, if feasible, were planned using Review Manager (RevMan v5.4) software. However, due to substantial heterogeneity in methodology, materials, and outcomes across the included studies, a meta-analysis was not performed in the present systematic review.

Results

**Figure 1:**
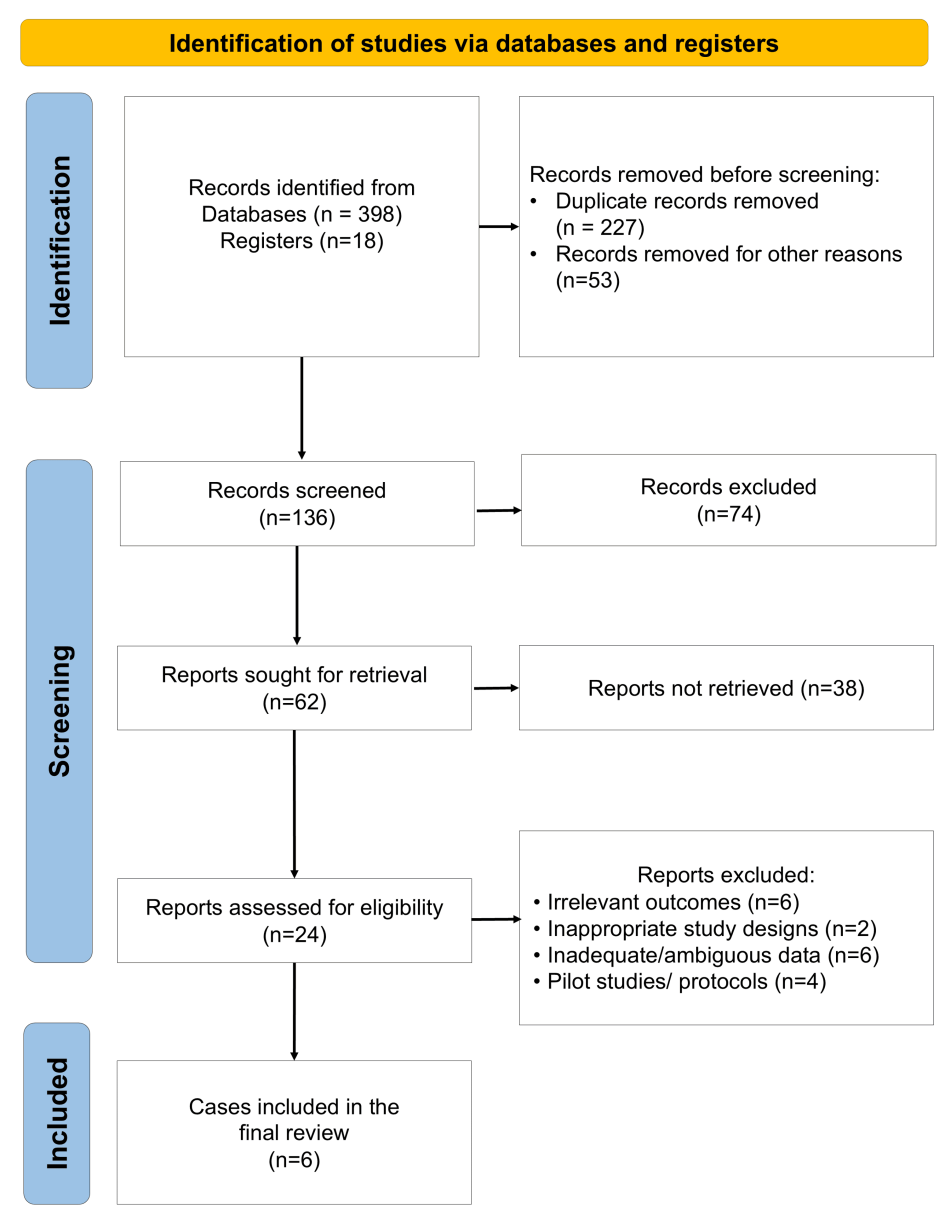
PRISMA flow diagram indicating the selection process of the articles in the present systematic review

A comprehensive electronic and manual search across databases yielded six eligible studies that met the predefined inclusion criteria for this systematic review (Figure 1) [27-32]. All selected studies directly compared inlay and onlay grafting techniques using autogenous or xenogeneic bone grafts for vertical and/or horizontal ridge augmentation in partially edentulous patients. These studies employed clinical and radiographic assessment tools, primarily cone-beam computed tomography (CBCT), to quantify changes in alveolar ridge dimensions, graft resorption, and healing outcomes over time. The use of CBCT for outcome assessment in the included studies is supported by its high diagnostic accuracy and three-dimensional measurement capability. Previous validation studies have demonstrated that CBCT provides reliable and precise detection of alveolar bone defects, including dehiscence and fenestration, with superior accuracy compared to conventional two-dimensional imaging. This supports the validity of radiographic measurements used across the included studies [33]. The data extracted from these studies are comprehensively summarized in Table 1 [27-32].

**Table 1:** Summary of included studies on inlay versus onlay grafting techniques RCT: randomized controlled trial; CBCT: cone-beam computed tomography; MBL: marginal bone loss; yrs: years; M: male; F: female; mm: millimetres; cc: cubic centimetres; NS: not significant; 3D: three-dimensional; NR: not reported; CI: confidence intervals

Author, year	Study design / country	Sample (age, gender)	Defect type	Inlay intervention	Onlay comparator	Graft source / type	Follow-up	Primary outcome(s)	Secondary outcome(s)	Key quantitative results	Complications	Conclusion
Atef et al., 2019 [27]	RCT, Egypt	n = 20; 29–54 yrs; 9M/11F	Horizontal mandibular ridge defect	Autogenous interpositional cortico-cancellous block (symphysis)	Autogenous onlay cortico-cancellous block (symphysis)	Autogenous block (symphysis)	4 months	Bone width gain (CBCT)	Graft resorption; soft-tissue healing	Inlay: Bone width increased from 3.85 ± 0.6 mm preoperatively to 8.84 ± 0.54 mm at four months (mean gain 5.02 ± 0.8 mm; graft resorption 0.357 ± 0.29 mm; P = 0.08, not significant). Onlay: Bone width increased from 3.74 ± 0.83 mm to 7.37 ± 1.98 mm (mean gain 3.6 ± 2.2 mm; graft resorption 0.448 ± 0.27 mm; P = 0.0006). Between-group comparison: Greater bone width at four months in the inlay group (P = 0.046). CI: NR	Mostly uneventful; transient paresthesia (inlay); two complications (onlay)	Inlay grafting produced greater horizontal gain and less resorption.
El Zahwy et al., 2019 [28]	RCT, Egypt	n = 16; Inlay 31–48 yrs; onlay 24–47 yrs; 10M/6F	Vertical anterior maxilla	Autogenous interpositional block (sandwich technique) + simultaneous implant	Autogenous onlay block + simultaneous implant	Autogenous block (symphysis)	6 months	Vertical bone gain (CBCT)	Marginal bone loss; soft-tissue healing; implant success	Inlay: Mean vertical bone gain 3.34 ± 1.2 mm; crestal bone loss 1.65 ± 0.94 mm at six months. Onlay: Mean vertical bone change −0.02 ± 1.86 mm; crestal bone loss 4.77 ± 1.67 mm at six months. Between-group comparison: Inlay showed significantly greater vertical gain and lower crestal bone loss (P < 0.05). CI: NR	Uneventful (inlay); 3 dehiscences + 3 exposures + 2 graft losses (onlay)	The inlay technique showed significantly higher vertical gain and fewer complications.
Elsayed et al., 2024 [29]	RCT, Egypt	n = 14; 20–43 yrs; 8M/6F	Horizontal anterior maxilla	Interpositional block graft (symphysis; piezo-osteotomy)	Buccal onlay block graft (symphysis)	Autogenous cortico-cancellous block	6 months	Horizontal gain (CBCT at 2, 5, 10 mm levels)	Graft resorption; soft-tissue healing	Inlay: Mean horizontal bone gain at six months = 2.4 ± 0.3 mm; graft resorption = 0.6 ± 0.2 mm. Onlay: Mean horizontal bone gain at six months = 2.4 ± 1.2 mm; graft resorption = 1.4 ± 0.5 mm. Between-group comparison: onlay showed significantly higher initial bone gain at T0 (P = 0.027), but significantly greater graft resorption at six months (P = 0.003); final bone gain at six months was comparable between groups (P = 0.234). CI: NR	Uneventful healing in both groups	Both viable; inlay showed less resorption and better long-term stability.
Mounir et al., 2017 [30]	RCT, Egypt	n = 16; 25–53 yrs; 10M/6F	Vertical anterior maxilla	Xenograft block (sandwich technique)	Particulate xenograft + titanium mesh	Xenograft (block for inlay; particulate for onlay)	6 months	Vertical bone gain	Complications: implant success	Inlay: Mean percentage vertical bone gain at six months = 31.6% ± 22.5%. Onlay: Mean percentage vertical bone gain at six months = 20.7% ± 13.3%. Between-group comparison: no statistically significant difference between techniques (P = 0.2). CI: NR	Inlay uneventful; onlay dehiscence + mesh exposure	Inlay provided higher gain and fewer complications.
Cordaro et al., 2010 [31]	Prospective cohort, Italy	n = 16; 38–67 yrs; 12F/4M	3D atrophy posterior maxilla	Sinus lift + particulated mandibular bone	Autogenous block grafts (ramus/chin)	Autogenous mandibular block ± Bio-Oss/Bio-Gide	Mean 40 months	Vertical and horizontal gain; resorption; implant success	Adverse events: donor site morbidity	Horizontal augmentation: Mean width increased from <4 mm at baseline to 5.5 ± 1.3 mm immediately post-grafting and 4.3 ± 1.1 mm at four months, with mean horizontal resorption of 1.2 mm (21%) (P < 0.01). Vertical augmentation: Mean height increased to 3.2 ± 0.6 mm immediately post-grafting and reduced to 2.1 ± 0.3 mm at four months, with mean vertical resorption of 1.1 mm (34%) (P < 0.05). Implant outcomes: 49 implants placed with 100% survival at 32–48 months follow-up. CI: NR	Minor events: dehiscence (1), swelling (14), hematoma (4), transient pulp sensitivity (7)	Autogenous mandibular grafting achieved stable 3D reconstruction with 100% implant success.
Barone et al., 2017 [32]	Retrospective radiological, Italy	n = 20; 35.8–65.4 yrs; 5M/15F	Vertical atrophy posterior mandible	Equine xenograft block (interpositional)	Autogenous onlay block (iliac crest)	Inlay: equine xenograft; onlay: autogenous iliac crest	4 months (volume); 12 months (implant MBL)	Volumetric gain; resorption	Marginal bone loss; complications	Inlay: Mean vertical gain 6.0 mm; volumetric gain 0.40 cc; graft loss 35% (1.7 mm); implant marginal bone loss 0.8 ± 0.3 mm at 12 months; surgical success rate 93.8% (95% CI 81.9–100%). Onlay: mean vertical gain 7.4 mm; volumetric gain 0.69 cc; graft loss 29% (1.9 mm); implant marginal bone loss 1.3 ± 0.4 mm; surgical success rate 82.4% (95% CI 64.2–100%). Between-group comparison: Marginal bone loss was significantly higher in the onlay group (P = 0.0006).	Dehiscences: 3 onlay, 1 inlay; 1 mandibular fracture (inlay); transient paraesthesia in 15	Both produced significant bone gain; inlay showed slightly better implant MBL and surgical success (93.8% vs. 82.4%).

The studies were conducted in different countries, predominantly from Egypt (n = 4) and Italy (n = 2), reflecting both regional research interests and the availability of grafting protocols [27-32]. All studies were published between 2010 and 2024, demonstrating continued clinical focus on optimizing ridge augmentation strategies for implant-supported rehabilitation. The majority were RCTs (n = 4), while one was a prospective cohort study and another a retrospective radiographic study [27-32]. Regarding sample size, included studies involved a total of 102 patients, with individual studies ranging from 14 to 20 participants. Participant ages ranged from 20 to 67 years, with most studies reporting mean ages of 38 to 54 years. Gender distribution across studies was balanced, with a slight female predominance noted in the Italian cohorts.

The type of ridge defect varied among the studies. Horizontal ridge defects were evaluated in three studies, vertical ridge defects in two studies, and complex three-dimensional posterior maxillary atrophy in one study. The inlay grafting technique typically involves interpositional block grafts placed between buccal and palatal/lingual plates or within a segmental osteotomy, often stabilized with miniplates. The onlay grafting technique, in contrast, involved crestally placed block or particulate grafts, secured with screws or titanium mesh [27-32]. Regarding graft materials, five studies used autogenous grafts, most commonly harvested from the mandibular symphysis or ramus, while one study used xenogeneic equine block grafts in the inlay group and autogenous iliac crest grafts in the onlay group. The follow-up periods ranged from 4 to 12 months, with most studies evaluating changes at four or six months postoperatively and one study extending to one year of implant follow-up.

The primary outcome in all studies was bone gain, measured horizontally or vertically, depending on the defect [27-32]. The inlay group consistently demonstrated either equivalent or superior bone gain when compared to the onlay group. For instance, in Atef et al. (2019) and Elsayed et al. (2024), both inlay and onlay groups achieved comparable horizontal bone gains, but graft resorption was significantly lower in the inlay group [27,29]. Similarly, El Zahwy et al. (2019) and Mounir et al. (2017) reported that inlay techniques led to greater vertical bone gain and fewer postoperative complications, despite slightly more complex surgical protocols [28,30]. Cordaro et al. (2010), in a prospective cohort study, showed that both techniques achieved successful ridge reconstruction, though onlay grafts demonstrated greater resorption in both horizontal and vertical dimensions [31]. Barone et al. (2017) found no significant difference in overall volumetric bone loss between inlay xenografts and onlay iliac grafts. Still, the implant marginal bone loss was significantly lower in the inlay group, suggesting better long-term stability [32].

The secondary outcomes included graft resorption, soft tissue healing, and adverse events. Inlay grafts were associated with less resorption in four out of six studies, and with better soft tissue healing outcomes, such as fewer incidences of flap dehiscence, graft exposure, or donor site morbidity. Onlay grafts, while technically simpler in some contexts, were frequently associated with higher resorption rates, ranging from 21% to over 35% depending on graft type and location. Implant survival rates were high across all studies, with no statistically significant failures during follow-up.

 Post-operative complications were reported in all studies, with a clear trend favoring the inlay grafting technique for soft-tissue healing and graft stability. In El Zahwy et al. (2019) and Mounir et al. (2017), flap dehiscence and graft exposure were observed exclusively in the onlay groups, while the inlay groups healed uneventfully [28,30]. Atef et al. (2019) reported only one instance of transient paresthesia in the inlay group, which resolved within a month, whereas the onlay group experienced more handling-related complications [27]. Similarly, Elsayed et al. (2024) found no post-operative complications in either group, but the onlay group showed greater graft resorption, indirectly reflecting compromised healing stability [29]. In the prospective study by Cordaro et al. (2010), minor complications such as swelling, hematomas, and transient pulpal sensitivity were reported across both groups. However, one case of flap dehiscence occurred in an onlay-treated site [31]. Barone et al. (2017) recorded three flap dehiscences and one case of paraesthesia in the onlay group, compared to only one surgical complication in the inlay group [32]. Collectively, these findings suggest that inlay grafts are associated with fewer soft-tissue complications and improved postoperative healing outcomes, likely due to their protected placement within the host bone and reduced tension on the overlying soft-tissue flap [18].

Overall, the findings suggest that while both inlay and onlay grafting techniques are effective for ridge augmentation, the inlay technique may offer advantages in terms of graft stability, reduced resorption, and lower complication rates, particularly in vertical or combined defects. However, the heterogeneity in study designs, graft materials, and outcome measures underscores the need for further well-powered, long-term clinical trials to establish definitive clinical guidelines.

Risk-of-bias assessment

**Figure 2:**
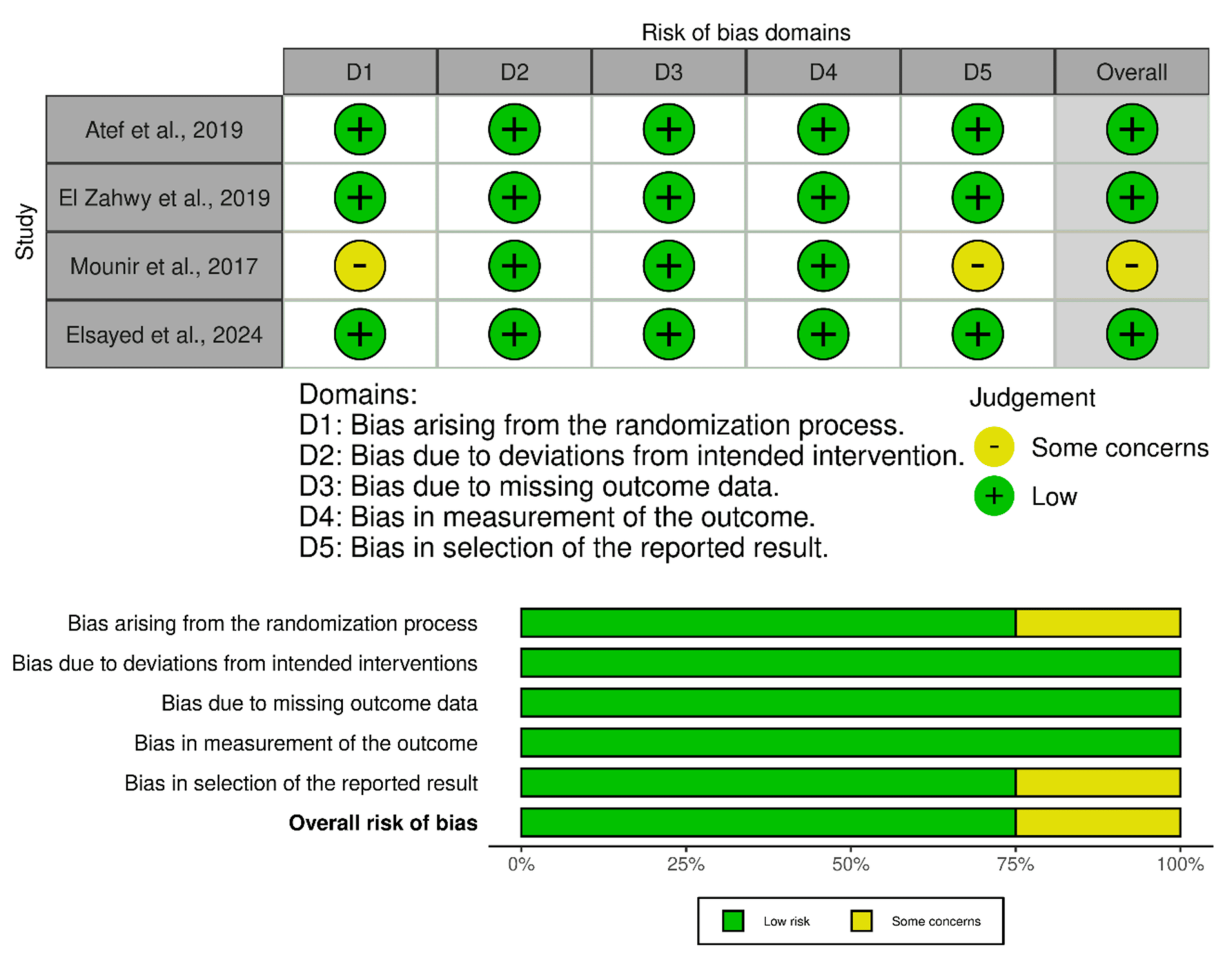
Risk of bias of the randomized controlled trials using the Cochrane RoB-2 tool [27-30]

Three studies, Atef et al. (2019), El Zahwy et al. (2019), and Elsayed et al. (2024), were assessed as having a low overall risk of bias [27-29]. These trials demonstrated a clear randomization process, precise adherence to assigned interventions, minimal missing outcome data, objective radiographic measurement of outcomes, and comprehensive reporting of prespecified results (Figure 2). Their methodological rigor lends strong internal validity to the findings reported, especially regarding horizontal and vertical bone gain following inlay versus onlay grafting procedures. The study by Mounir et al. (2017) was identified as having some concerns overall [30]. Although it adhered to intervention protocols and had no missing data, the randomization method was not clearly described, and selective reporting could not be ruled out because of limited detail on whether all intended outcomes were analyzed. This introduces uncertainty when interpreting the magnitude of bone gain and complication rates observed in that study.

The prospective cohort study by Cordaro et al. (2010) was evaluated using the ROBINS-I tool (Table 2) and found to have an overall moderate risk of bias [31]. The primary limitation was a lack of randomization and minimal statistical adjustment for potential confounding variables. While the selection of participants was clearly defined and the classification of interventions was well documented, outcome assessors were not blinded, increasing the potential for observational bias, especially in subjective parameters such as graft resorption and healing. Furthermore, although most outcomes were reported, not all variability or subgroup analyses were presented, raising concerns about selective reporting. Nevertheless, the low risk of missing data and adherence to intervention protocols support the overall reliability of the results, albeit with some caution in interpretation.

**Table 2:** Risk of bias of the non-randomized controlled trials using the ROBINS-I tool

Domain	Cordaro et al., 2010 [31]
Bias due to Confounding	Moderate - no randomization, limited control for confounders
Bias in the Selection of Participants	Low - clear eligibility criteria and consecutive sampling
Bias in the Classification of Interventions	Low - interventions clearly defined
Bias due to Deviations from Intended Interventions	Low - interventions delivered as planned
Bias due to Missing Data	Low - minimal missing data
Bias in the Measurement of Outcomes	Moderate - assessors not blinded
Bias in the Selection of the Reported Result	Moderate - not all outcome variability discussed
Overall Risk of Bias	Moderate

On the other hand, Barone et al. (2017) assessed using the Newcastle-Ottawa Scale received a total score of 5 out of 9, corresponding to a moderate-quality rating (Table 3) [32]. The study performed well in the selection domain, as it utilized secure records and clearly identified representative patient cohorts with well-documented baseline characteristics. However, it failed to control for confounding variables, which is a significant limitation in a retrospective study design. Although outcomes were measured using radiographic analysis, an objective and reliable method, the retrospective nature of data collection introduces potential for recall and measurement biases. Furthermore, the absence of standardized criteria for evaluating some outcome parameters reduced the comparability and reproducibility of the findings.

**Table 3:** Risk of bias of the cross-sectional studies by the Newcastle-Ottawa Scale

Domain	Barone et al., 2017 [32]
Selection (max 4)	3 - Representative sample, secure records, outcome not present at start
Comparability (max 2)	0 - No control for confounders
Outcome (max 3)	2 - Radiographic follow-up used, sufficient duration
Total Score	5
Quality Rating	Moderate

Certainty of evidence

Using the Grading of Recommendations Assessment, Development, and Evaluation (GRADE) approach [34], the certainty of evidence was evaluated across six studies (n = 102) comparing inlay versus onlay ridge augmentation (Table 4) [27-32]. Owing to substantial heterogeneity in defect types, graft materials, surgical protocols, and outcome reporting (including limited availability of 95% confidence intervals), results were synthesized narratively, and evidence was downgraded primarily for imprecision, and where relevant for inconsistency and indirectness. Publication bias could not be formally assessed because only six studies were available, and a minimum of ten are required to generate a valid funnel plot.

**Table 4:** GRADE certainty of evidence (summary of findings) for inlay versus onlay ridge augmentation (six studies; total n = 102) CBCT: cone-beam computed tomography; CI: confidence interval; MBL: marginal bone loss; mm: millimetre(s); %: percentage; RCT: randomized controlled trial

Outcome (follow-up)	Studies contributing (design)	Participants	Direction of the effect (inlay vs. onlay)	Certainty of evidence	Main reasons for rating down
Horizontal ridge width gain (four to six months; CBCT)	2 (RCTs: Atef, 2019; Elsayed, 2024)	34	Uncertain difference in the final width gain (one RCT favored inlay; one found comparable final gain), but the trend favors inlay for dimensional stability	Moderate	Imprecision (small samples; CIs not reported); inconsistency in the magnitude of the width gain between trials
Vertical ridge height gain (six months; CBCT/percent gain)	2 (RCTs: El Zahwy, 2019; Mounir, 2017)	32	Inconsistent: one RCT showed clearly greater vertical gain with inlay; the other showed no statistically significant difference	Very low	Risk of bias (some concerns in one RCT), inconsistency (discordant results), imprecision (small samples; CIs not reported; outcome metrics not harmonized)
Graft resorption / graft loss (hard-tissue stability) (four to six months; radiographic)	4 (2 RCTs + 2 non-randomized: Cordaro, 2010; Barone, 2017)	70	Generally favors inlay (less resorption in both horizontal RCTs; observational studies broadly support stability advantages, although metrics vary)	Low	Study limitations (non-randomized designs contribute), indirectness/inconsistency of measurement (mm, %, volumetric; different sites/materials), imprecision (CIs largely not reported)
Soft-tissue complications (dehiscence/exposure/graft loss) (four to six months; clinical)	6 (4 RCTs + 2 non-randomized)	102	The trend favors inlay (complications are more frequent in onlay in multiple studies), but event reporting is non-uniform.	Low	Risk of bias (non-randomized evidence; limited blinding), imprecision (few events; incomplete denominators), inconsistency in definitions/reporting across studies
Implant-related outcomes (crestal/ marginal bone loss; survival/success) (six to 12 months for MBL; longer survival reported in one cohort)	4 (2 RCTs + 2 non-randomized: Cordaro, 2010; Barone, 2017)	68 (implant counts variably reported)	Favors inlay for peri-implant bone maintenance in some studies; overall survival appears high, but comparability is limited.	Low	Risk of bias (non-randomized designs), indirectness (different implant timing/protocols and graft materials), imprecision (small samples; limited CI reporting), inconsistency in outcome definitions/timepoints

Certainty was moderate for horizontal ridge width gain because the two RCTs showed clinically meaningful gains in both groups, with limited precision due to small samples and absent confidence intervals [27,29]. Certainty was very low for vertical ridge height gain because the RCTs reported discordant findings and used non-harmonized metrics, limiting confidence in comparative effects [28,30]. Evidence was low for hard-tissue stability (resorption/graft loss) and soft-tissue complications: short-term RCT data [27-30] and non-randomized studies [31,32] generally suggested better stability and fewer soft-tissue events with inlay, but certainty was reduced by heterogeneous measurement approaches and inconsistent event reporting. Certainty for implant-related outcomes was also low, as these endpoints were variably defined and timed across four studies [28,30-32], and were influenced by differences in implant timing and follow-up. Therefore, while the direction of effect often favors inlay for stability and peri-implant bone maintenance, robust standardized RCTs are needed to confirm the magnitude and durability of benefit.

Discussion

The findings of the present systematic review offer meaningful insights into the comparative performance of inlay and onlay grafting techniques for vertical and horizontal ridge augmentation in atrophic alveolar ridges. Across the included studies, both methods consistently resulted in appreciable improvements in bone volume; however, the inlay technique demonstrated more favorable outcomes in maintaining augmented bone, minimizing graft resorption, and reducing post-operative complications [27-32]. These observations are clinically significant, especially in implant dentistry, where achieving stable and sufficient bone volume is essential for long-term implant success [35]. The reviewed evidence indicates that the biological and mechanical advantages of the inlay approach may provide superior conditions for graft stability and integration.

Inlay grafting, also known as the interpositional graft or sandwich osteotomy technique, involves inserting a graft block within a prepared osteotomy between the remaining alveolar bone segments [36]. This configuration ensures intimate contact between the graft and host bone on multiple surfaces, facilitating enhanced revascularization and superior mechanical support during healing [37]. These biological attributes likely account for the superior clinical performance repeatedly observed in the included studies [38]. El Zahwy et al. and Mounir et al. both documented significantly greater vertical bone gain in the inlay groups, with less crestal bone loss and fewer soft-tissue complications than in the onlay groups [28,30]. The protected positioning of the graft within the host bone reduces its exposure to mechanical loading, flap tension, and periosteal pressure, which are factors commonly associated with graft resorption in onlay placements [39]. Additionally, the sandwich osteotomy can allow simultaneous implant placement in selected cases, reducing overall treatment time and improving patient convenience [40].

Interestingly, the biological advantages observed with inlay grafting techniques parallel findings reported in other bone-supportive surgical interventions, such as periodontally accelerated osteogenic orthodontics (PAOO). Systematic evidence suggests that controlled corticotomies and interpositional grafting in PAOO not only accelerate tooth movement but also help preserve alveolar bone thickness and periodontal support [41]. These outcomes are attributed to enhanced regional acceleratory phenomena and improved vascular supply, mechanisms that closely resemble those facilitating graft stability in inlay ridge augmentation. Such parallels reinforce the concept that surgically protected graft positioning and enhanced vascular access are key determinants of successful alveolar bone regeneration [41].

In contrast, the onlay grafting technique, while widely used and often more straightforward, showed higher rates of graft resorption and post-operative complications across several studies, even in osseous structures other than the jawbones [42,43]. Onlay grafts primarily rely on revascularization from the underlying recipient bed and have limited vascular contact surfaces compared with inlay grafts [19,44]. This reduced blood supply may contribute to delayed integration and greater volumetric shrinkage during healing, particularly in vertically augmented areas, where gravitational forces and soft-tissue pressure can destabilize the graft [45]. Studies by Atef et al. and Elsayed et al. revealed that although initial bone gains were similar between the techniques, the inlay groups maintained significantly greater augmented volume over time. In contrast, the onlay grafts exhibited pronounced post-healing resorption [27-29]. Cordaro et al. further supported these findings, reporting that although onlay grafts provided immediate dimensional enhancement, a substantial proportion of the achieved volume was lost by the time of implant placement [31].

Soft tissue response also emerged as a key differentiating factor between the two methods. Because the inlay graft is contained within the bony envelope, it does not exert pressure on the overlying soft tissues. It therefore presents a lower risk of flap tension, dehiscence, or graft exposure [46]. This contrast was evident in studies by El Zahwy et al. and Mounir et al., in which onlay procedures were associated with multiple instances of flap breakdown, exposure, and partial graft loss. In contrast, the inlay groups healed with fewer complications [28,30]. Such complications not only jeopardize graft stability but also increase patient morbidity and can delay subsequent implant placement [47]. Inlay grafts, by comparison, demonstrated a generally lower incidence of adverse outcomes and improved implant survival following augmentation [33,48].

A noteworthy study by Barone et al. compared autogenous onlay iliac crest grafts with equine xenogenic inlay grafts and reported no significant difference in volumetric gain; however, the inlay group exhibited significantly lower marginal bone loss around implants after one year [32]. Despite one mandibular fracture during osteotomy, surgical success remained higher in the inlay group, suggesting that the mechanical protection afforded by the inlay position may support long-term stability even when non-autogenous grafts are used [49].

Post-operative complications across the included studies showed a consistent trend favoring the inlay approach. The most common complications associated with onlay grafts were flap dehiscence, graft exposure, and partial or total graft loss, likely due to increased soft tissue tension and compromised vascularity at the graft site [16,20,50]. In contrast, inlay grafts were associated with uneventful healing in most cases because they are protected within the osteotomized site and benefit from enhanced blood supply [37,51]. Atef et al. reported only one case of transient paresthesia in the inlay group, while the onlay group showed multiple complications [27]. Even in more demanding clinical scenarios described by Cordaro et al. and Barone et al., healing was largely favorable with minimal adverse events in the inlay groups, and implant survival remained high [31,32]. Collectively, these findings support the biomechanical and biological superiority of inlay grafts in anatomically challenging ridges.

Although this systematic review's findings highlight a clear advantage of the inlay technique, several limitations must be acknowledged. Only a small number of high-quality RCTs directly compared these techniques, and the remaining evidence included non-randomized studies that were more susceptible to confounding. Considerable heterogeneity existed among studies regarding surgical technique, graft material, defect morphology, and outcome measurement. Many studies lacked long-term follow-up, limiting assessment of the durability of augmented bone and the long-term success of implants placed in regenerated bone. Incomplete reporting of complications and variation in radiographic measurement protocols further restricted comparability and generalizability. Future research should focus on larger, multicenter randomized trials with standardized protocols, longer follow-up, and advanced imaging modalities to better characterize outcomes. Additionally, evaluating novel biomaterials, minimally invasive techniques, and patient-centered outcomes, such as satisfaction and cost-effectiveness, would enhance clinical decision-making. A deeper understanding of graft biology and host response mechanisms will support improved surgical refinements and predictable ridge augmentation outcomes.

## Conclusions

The findings of this systematic review indicate that while both inlay and onlay grafting techniques are capable of achieving clinically meaningful vertical and horizontal ridge augmentation, the inlay approach consistently demonstrates superior stability, reduced graft resorption, and more favorable soft-tissue healing in most clinical scenarios. These advantages appear particularly relevant in vertical augmentation cases, where biomechanical and vascular considerations are critical to successful outcomes. Nevertheless, the choice of technique should remain defect-specific, taking into account anatomical constraints, surgical expertise, and prosthetic objectives. Further high-quality randomized controlled trials with standardized methodologies and long-term follow-up are essential to validate these findings, refine clinical protocols, and establish evidence-based guidelines for optimizing alveolar ridge augmentation.

## Appendices

**Table 5:** Database-specific search strategies

Database (interface)	Search string	Limits applied
PubMed (NCBI)	(("Alveolar Ridge Augmentation"[MeSH Terms] OR "Alveolar Bone Loss"[MeSH Terms] OR "Alveolar Process"[MeSH Terms] OR alveolar ridge augment*[tiab] OR ridge augment*[tiab] OR ridge reconstruct*[tiab] OR bone augment*[tiab]) AND ("Bone Transplantation"[MeSH Terms] OR "Bone Regeneration"[MeSH Terms] OR "Dental Implantation, Endosseous"[MeSH Terms] OR "Dental Implants"[MeSH Terms] OR autogenous[tiab] OR autologous[tiab] OR autograft*[tiab] OR "bone block"[tiab] OR block graft*[tiab]) AND (inlay[tiab] OR onlay[tiab] OR interpositional[tiab] OR "sandwich technique"[tiab] OR "sandwich osteotomy"[tiab] OR "bone block graft"[tiab]))	Humans; English; no date limits
MEDLINE (Ovid)	1. exp Alveolar Ridge Augmentation/ OR exp Alveolar Bone Loss/ OR Alveolar Process/ 2. (alveolar ridge augment* OR ridge augment* OR ridge reconstruct* OR bone augment*).ti,ab,kf. 3. exp Bone Transplantation/ OR exp Bone Regeneration/ OR exp Dental Implantation, Endosseous/ OR Dental Implants/ 4. (autogenous OR autologous OR autograft* OR “bone block” OR block graft*).ti,ab,kf. 5. (inlay OR onlay OR interpositional OR “sandwich technique” OR “sandwich osteotomy”).ti,ab,kf. 6. (1 OR 2) AND (3 OR 4) AND 5 7. limit 6 to (humans AND english language)	Humans; English; no date limits
Embase	1. 'alveolar ridge augmentation'/exp OR 'alveolar bone loss'/exp OR 'alveolar process'/exp 2. (alveolar ridge augment* OR ridge augment* OR ridge reconstruct* OR bone augment*).ti,ab,kw. 3. 'bone transplantation'/exp OR 'bone regeneration'/exp OR 'dental implantation'/exp OR 'dental implant'/exp 4. (autogenous OR autologous OR autograft* OR 'bone block' OR block graft*).ti,ab,kw. 5. (inlay OR onlay OR interpositional OR 'sandwich technique' OR 'sandwich osteotomy').ti,ab,kw. 6. (1 OR 2) AND (3 OR 4) AND 5 7. limit 6 to (human AND english language)	Humans; English; no date limits
EBSCOhost	((MH "Alveolar Ridge Augmentation" OR MH "Alveolar Bone Loss" OR MH "Alveolar Process" OR TI (alveolar ridge augment* OR ridge augment* OR ridge reconstruct* OR bone augment*) OR AB (alveolar ridge augment* OR ridge augment* OR ridge reconstruct* OR bone augment*)) AND (MH "Bone Transplantation" OR MH "Bone Regeneration" OR MH "Dental Implantation, Endosseous" OR MH "Dental Implants" OR TI (autogenous OR autologous OR autograft* OR "bone block" OR block graft*) OR AB (autogenous OR autologous OR autograft* OR "bone block" OR block graft*)) AND (TI (inlay OR onlay OR interpositional OR "sandwich technique" OR "sandwich osteotomy") OR AB (inlay OR onlay OR interpositional OR "sandwich technique" OR "sandwich osteotomy")))	Humans (if available); English; no date limits
Cochrane CENTRAL (Cochrane Library)	#1 MeSH descriptor: [Alveolar Ridge Augmentation] explode all trees #2 MeSH descriptor: [Bone Transplantation] explode all trees #3 MeSH descriptor: [Bone Regeneration] explode all trees #4 (alveolar ridge augment* OR ridge augment* OR ridge reconstruct* OR bone augment*):ti,ab,kw #5 (autogenous OR autologous OR autograft* OR "bone block" OR block graft*):ti,ab,kw #6 (inlay OR onlay OR interpositional OR "sandwich technique" OR "sandwich osteotomy"):ti,ab,kw #7 (#1 OR #4) AND (#2 OR #3 OR #5) AND #6	No date limits; English language
Web of Science Core Collection	TS=((alveolar NEAR/2 ridge NEAR/2 augment*) OR (ridge NEAR/2 augment*) OR (ridge NEAR/2 reconstruct*) OR (bone NEAR/2 augment*)) AND TS=((inlay OR onlay OR interpositional OR "sandwich technique" OR "sandwich osteotomy")) AND TS=((autogenous OR autologous OR autograft* OR "bone block" OR "block graft*"))	Document types: Article/Clinical Trial (optional); English; no date limits
Google Scholar	("alveolar ridge augmentation" OR "ridge augmentation" OR "ridge reconstruction" OR "bone augmentation") AND (inlay OR interpositional OR "sandwich technique" OR "sandwich osteotomy" OR onlay) AND (autogenous OR autologous OR autograft OR "bone block" OR "block graft")	No date limits; sorted in relevance order
